# Enhanced prediction of microvascular invasion in hepatocellular carcinoma: a comparative study of intraoperative ultrasound, CT imaging and radiomic

**DOI:** 10.1007/s11547-026-02211-2

**Published:** 2026-04-29

**Authors:** Matteo Barabino, Francesco Rizzetto, Gaetano Piccolo, Elisa Rondi, Silvia Tortora, Claudia Cigala, Umberto Gianelli, Roberto Santambrogio, Paolo Pietro Bianchi

**Affiliations:** 1 General Surgery Unit, Department of Health Sciences (DISS), San Paolo Hospital, University of Milan, Via Antonio Di Rudinì 8, 20142 Milan, Italy; 2 Department of Radiology, ASST Grande Ospedale Metropolitano Niguarda, Milan, Italy; 3 Department of Diagnostic Radiology, San Paolo Hospital, Milan, Italy; 4 Pathology Unit, Department of Health Sciences (DISS), San Paolo Hospital, University of Milan, Milan, Italy; 5 Unit of Hepato-Biliary Surgery, Unit of General Surgery, ASST Fatebenefratelli Sacco, Milan, Italy

**Keywords:** Radiomic, Intraoperative ultrasound, Hepatocellular carcinoma, CT imaging, Microvascular invasion

## Introduction

Hepatocellular carcinoma (HCC) is the most common primary liver malignancy, accounting for nearly 90% of cases worldwide [1]. Despite improvements in surgical strategies and perioperative care, the five-year recurrence rate following hepatic resection (HR) remains at approximately 70%, even in early-stage HCC [2, 3]. This disappointing outcome is often linked to aggressive pathological features, including satellite nodules, microvascular invasion (MVI) and capsule infiltration [4], collectively defining microinvasive HCC (MI-HCC).

Vascular invasion, both macro- and microvascular, is a key prognostic factor associated with early recurrence and reduced survival after HCC treatment [5–7]. Although macrovascular invasion can be identified through standard preoperative imaging, which informs proper staging and therapeutic decision-making [8], MI-HCC can only be confirmed postoperatively via histopathological assessment. Current preoperative imaging modalities, such as CT and MRI, have shown limited success in detecting MI-HCC, because its signs are challenging to identify and not unanimously recognized [7, 9]. In contrast, intraoperative ultrasound (IOUS) has emerged as a valuable technique, demonstrating approximately 85% accuracy in identifying adverse prognostic factors such as MI-HCC [10]. Nevertheless, IOUS requires specialized expertise, and both CT and MRI rely on subtle contrast differences between tumors and adjacent tissues that can be challenging to interpret [6].

Over the past decade, the recognition that deeper, quantitative data within medical images can provide far more informative insights into tumor characteristics than what is visible to the human eye has grown exponentially [5]. By leveraging sophisticated algorithms and statistical techniques, radiomics can identify predictive and prognostic imaging features that correlate with tumor biology. Recent studies applying radiomics to detect MI-HCC have shown encouraging results, reporting high accuracy rates and AUC values between 0.76 and 0.98 [11–15]. However, its clinical adoption has been slowed by the lack of biological interpretability, which hinders the translation of radiomic findings into clinical practice.

In this context, this double-blind, retrospective study—the first to directly compare IOUS, conventional CT features and CT-derived radiomics—aims to assess the diagnostic accuracy of preoperative imaging in detecting MI-HCC and to determine whether radiomics-based texture analysis can reveal distinctive patterns associated with this condition. Ultimately, the goal is to develop a robust, reproducible model that enhances the preoperative detection of MI-HCC, enabling more personalized and effective treatment strategies.

## Material and methods

### Study design and patient population

This single-center retrospective observational study was approved by the local Ethics Committee, with informed consent waived because of the retrospective collection of anonymized data. The study was conducted in compliance with the Declaration of Helsinki and adhered to the Checklist for Evaluation of Radiomics Research (CLEAR) guidelines [16].

All adult patients (≥ 18 years) who underwent HR for histologically confirmed HCC lesions were included if they had a preoperative CT scan within three months prior to HR and available IOUS data.

The following exclusion criteria were applied: 1) HCC nodules < 10 mm on preoperative CT; 2) HCC nodules previously treated with nonsurgical methods, including percutaneous or laparoscopic thermal ablation, alcohol injection, transarterial chemoembolization/radioembolization; 3) incomplete or inadequate CT scans (e.g., improper contrast timing, severe artifacts affecting image quality); 4) macrovascular invasion on preoperative CT imaging; and 5) intraoperative complications that could interfere with IOUS evaluation or compromise the validity of the collected data.

For all patients, demographic information (age, sex) and clinical data, including body mass index (BMI), hepatopathy etiology, Child–Pugh class, model for end-stage liver disease (MELD) score, Barcelona Clinic Liver Cancer (BCLC) stage, diabetes status, presence of esophageal varices and any prior treatment for HCC, were collected. Additionally, preoperative blood test results, including platelet count and total bilirubin, aspartate aminotransferase (AST), alanine aminotransferase (ALT), cholinesterase (CHE), alkaline phosphatase (ALP) and alpha-fetoprotein (AFP) levels, were recorded.

### Preoperative CT protocol and radiological evaluation

The CT scans varied in protocol, as many patients were referred to our institution from external hospitals where preoperative imaging had been performed. For eligibility, CT images were required to include axial reconstructions of the late arterial phase, portal venous phase and delayed phase, acquired in accordance with the technical recommendations of the Liver Imaging Reporting and Data System (LI-RADS) guidelines [17], insofar as this could be verified.

A radiologist with eight years of experience in hepatobiliary imaging, aware that all patients had HCC but who was blinded to the clinical, IOUS and pathological data, retrospectively evaluated the CT images. The radiologist categorized the CT patterns based on the following criteria: tumor diameter (either ≤ 2 cm or not); vascularity (hypervascular in the arterial phase or not hypervascular); homogeneity (homogeneous or inhomogeneous); nodular appearance (well-defined or irregular margins); capsular interruption; mosaic appearance; nodule-in-nodule appearance; satellites (i.e., small nodules within 2 cm of the main HCC nodule); and high-risk signs of vascular/biliary invasion (encasement or close contact between vascular/biliary structures and nodule margins).

### IOUS evaluation

The IOUS examinations were performed by two surgeons with over ten years of experience who were working independently and were blinded to the pathological findings. In cases of discrepancies, the ultrasound findings were reviewed and evaluated collectively to reach a consensus.

Patients were classified based on HCC characteristics identified by IOUS[8], including tumor diameter (either ≤ 2 cm or not), echogenicity (iso-/hyperechoic or hypoechoic), ultrasound texture (homogeneous or inhomogeneous), nodular appearance (well-defined nodular shape or irregular margins), capsular interruption, mosaic architecture, nodule-in-nodule appearance, satellites (i.e., small nodules ≤ 2 cm from the main HCC lesion) and high-risk signs of vascular/biliary invasion (encasement or close contact between vascular/biliary structures and nodule margins).

Details about the IOUS setup are reported in Supplementary Materials.

### Radiomic analysis

**Fig. 1:**
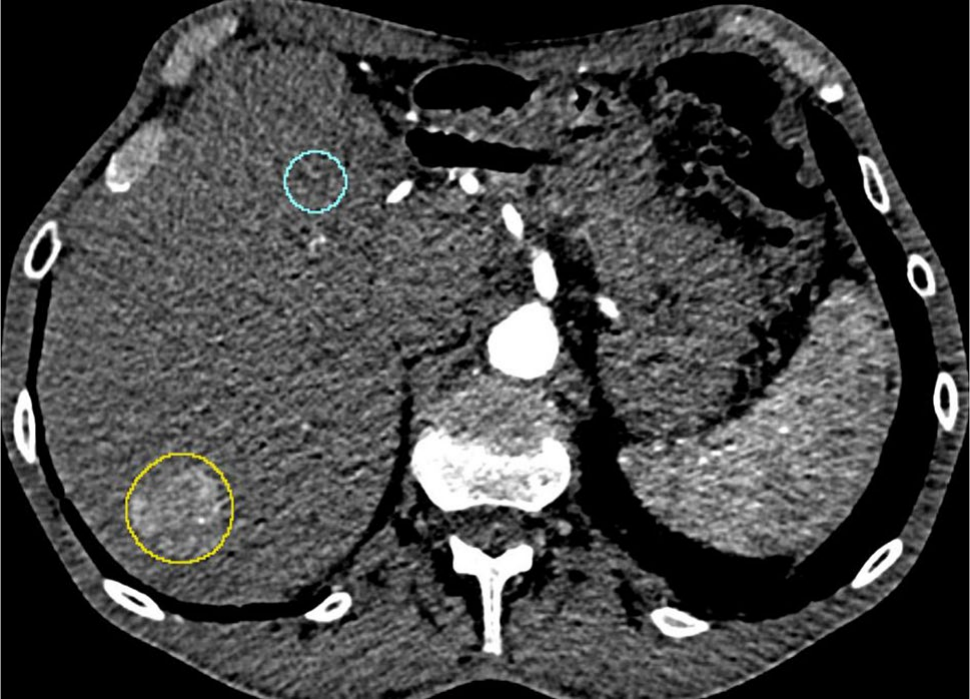
Case example of segmentation of HCC (yellow circle) and background liver parenchyma (blue circle)

Radiomic analysis was conducted by a radiologist with six years of experience in quantitative abdominal imaging research. Using the image analysis platform 3D Slicer v5.6.2 (www.slicer.org), HCC nodules were contoured on arterial, portal venous and delayed phases to segment the entire lesion volume. If the HCC was not visible in one of the contrast phases, segmentation was performed after co-registering it with a phase in which the tumor was clearly visible. A 3-mm rim of peritumoral parenchyma was automatically added to the lesion segmentation and manually corrected to ensure the exclusion of vessels and nonhepatic structures. Additionally, a 2 cm circular region of background liver parenchyma was segmented in the lobe opposite the lesion or, if this was not feasible, in the most distant area from all visible tumors. All significant hepatic vessels were excluded from the segmentation process. The segmentations (Fig. 1) were used to extract 107 radiomic features (RFs) using the PyRadiomics Python package (v3.10), a tool adhering to the Image Biomarker Standardizations Initiative (IBSI) standard [18].

Prior to feature extraction, gray levels were discretized using a fixed bin number of 20 over a predefined intensity range of 340 Hounsfield units (− 19 to 320 HU), and images were resampled to isotropic voxels of 3 mm to reduce inter-scan variability and improve dataset homogeneity.

All RFs were extracted from the CT images of each patient across all contrast phases using two methods: directly from the tumor and normalized to the background of the liver. Normalization was performed by dividing the value of each feature extracted from the tumor by the corresponding feature value extracted from the liver background in the same contrast phase.

Full details of the radiomic analysis are reported in the Supplementary Materials.

### Histopathological evaluation

A pathologist with over ten years of experience in hepatobiliary pathology conducted a blinded evaluation of the surgical samples, without knowledge of the IOUS or CT examination results. In accordance with the histological definition proposed by Yamashita et al.[4], MI-HCC was defined as a tumor exhibiting vascular/biliary microinvasion (the presence of tumor cells in vascular or biliary spaces discernible only under microscopic examination [19]) and/or the presence of satellites (tumor cells located around or near the main lesion but separated from it, with histological characteristics similar to those of the primary tumor [20]).

### Data analysis

Categorical variables are expressed as counts and percentages with 95% confidence intervals (95% CIs) using the Wilson method. Continuous variables are reported as medians with interquartile ranges (Q1–Q3). Continuous variables were compared within groups using the Wilcoxon matched-pairs test and between groups using the Mann‒Whitney U test. Categorical variables were analyzed using Pearson’s Chi-square test.

The histological report was used as the ground truth for the analysis. Mirroring Yamashita’s histological definition [4], MI-HCC on preoperative CT and IOUS was determined by the presence of vascular/biliary infiltration and/or satellite nodules, as above defined for each imaging modality.

Independent risk factors for MI-HCC on CT and IOUS were identified using logistic regression.

For radiomic modeling, outcome-stratified randomization was performed to divide into 70% training–validation (80:20 split) and 30% independent test cohorts. To reduce dimensionality and overfitting, Spearman’s correlation (ρ > 0.90) was used to exclude redundant RF. The remaining RFs were normalized and subjected to an additional selection process via least absolute shrinkage and selection operator (LASSO) regression [21]. Automatic hyperparameter tuning was employed to optimize the alpha parameter in LASSO regression by minimizing the average Akaike information criterion during fivefold stratified cross-validation with shuffling [22]. To limit overfitting, a maximum number of features was set according to the “rule of thumb” of maintaining a 1:10 ratio between the number of variables and the sample size [23].

The selected RFs, derived from both direct and normalized extractions, were used to train and validate support vector machine (SVM)[24] models for each contrast phase, with the presence of MI-HCC as the outcome. SVM was chosen because of its well-documented stability in small- to medium-sized datasets and suitability for high-dimensional feature spaces typical of radiomic analyses [25]. When multiple models were available, the model with the best performance was selected and applied to the independent test cohort. Additionally, the CT and IOUS variables with a *p* value < 0.10 in the logistic regression analysis were used to train and validate separate SVM models, which were subsequently applied to the same independent test cohort.

For all the models, the performance in identifying MI-HCC was evaluated in terms of sensitivity (SE), specificity (SP), positive likelihood ratio (PLR), negative likelihood ratio (NLR) and accuracy (ACC). The likelihood ratios were interpreted as recommended by Jaeschke et al.[26]. Receiver operating characteristic (ROC) curves were also constructed to estimate the diagnostic performance in identifying MI-HCC, with corresponding areas under the curve (AUCs) and 95%CIs calculated. The AUC values in the test population were compared using DeLong’s test [27].

Data analysis was performed using the Real Statistics Resource Pack software (Release 6.8) (www.real statistics.com) for Microsoft Excel (Microsoft Corporation, Redmond, Washington, USA), GraphPad Prism 9.5.1 (GraphPad Software, La Jolla, CA) and Python v3.10.

For all analyses, statistical significance was established at *p* < 0.050.

## Results

**Fig. 2:**
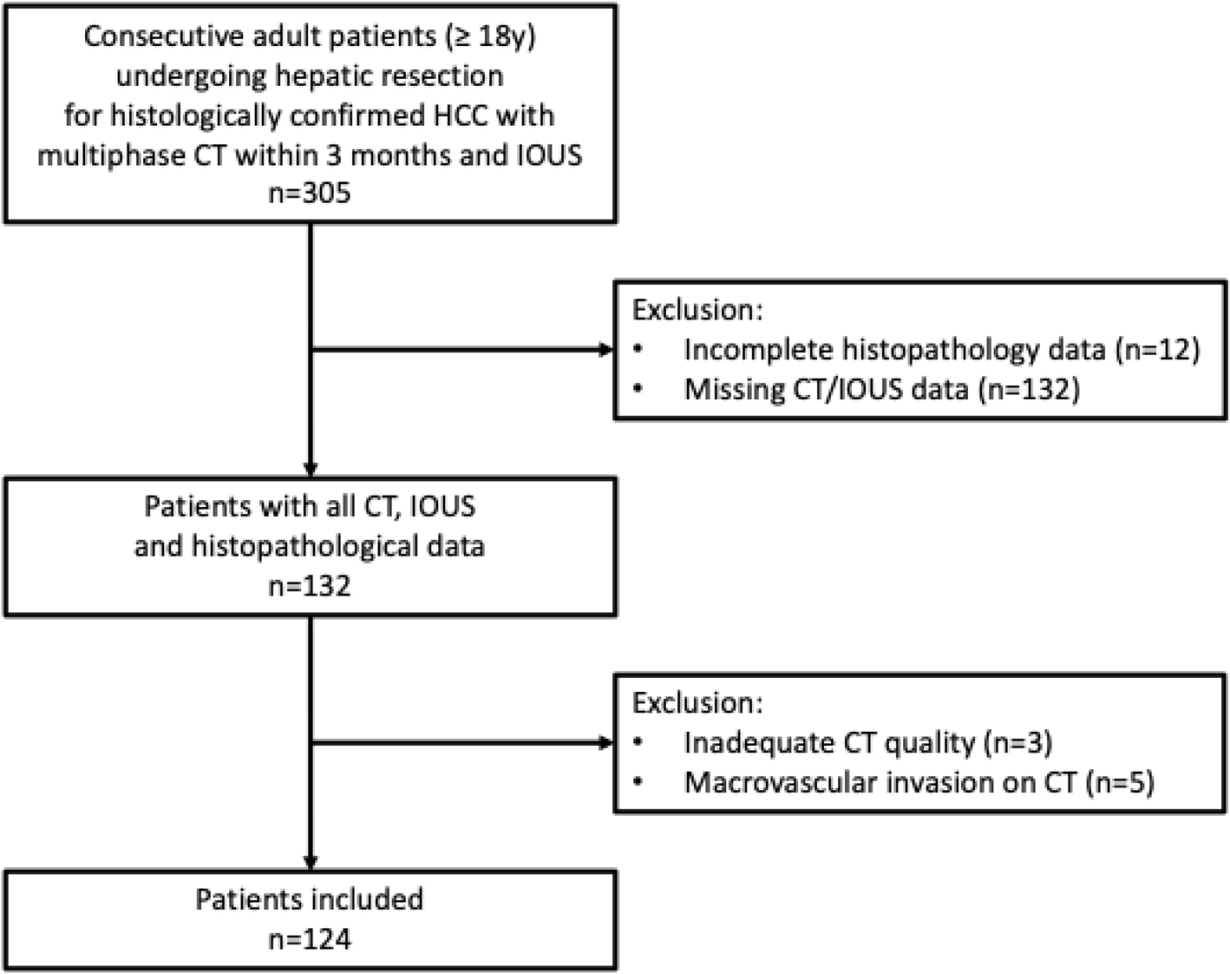
Flowchart of patient selection

A total of 305 patients with HCC who underwent HR between December 2008 and June 2023 were initially considered for inclusion. Among these, 12 patients were excluded because of incomplete pathology data. An additional 164 patients were removed because of inadequate or missing CT or IOUS data, and 5 more patients were excluded because of the presence of macrovascular invasion on CT images. Only one of the included patients (1%) had more than one HCC nodule (specifically, two) for which both IOUS and pathological data were available. Although the analysis in this study was lesion-based, we opted to include only the largest of the two nodules, as both shared identical CT, IOUS and histopathological features. Consequently, the final study cohort comprised 124 patients with 124 analyzed HCC nodules (Fig. 2). The demographics and clinical characteristics of patients are reported in Table 1.

**Table 1:** Baseline characteristics of the study population, stratified by groups. Data are presented as count and percentage or median with 25th and 75th percentiles, as appropriate

Variable	All patients (n = 124)	Training–validation (n = 86)	Test (n = 38)
Male sex (%)	92 (74%)	66 (77%)	26 (68%)
Age (y)	70 (60–75)	68 (59–74)	73 (63–75)
Cirrhosis etiology (%)	118 (95%)	85 (99%)	33 (87%)
HCV	77 (62%)	55 (64%)	22 (58%)
HBV	29 (23%)	21 (24%)	8 (21%)
Alcohol	25 (23%)	20 (23%)	5 (13%)
MASH	10 (8%)	7 (8%)	3 (8%)
Other	7 (6%)	5 (6%)	2 (5%)
Mixed etiology	29 (23%)	22 (26%)	7 (18%)
Noncirrhotic (%)	6 (5%)	1 (1%)	5 (13%)
Child–Pugh (%)			
Class A5	102 (82%)	67 (76%)	35 (92%)
Class A6	21 (17%)	18 (21%)	3 (8%)
Class B7	1 (1%)	1 (1%)	0 (0%)
MELD score	8 (7–9)	8 (7–9)	7 (7–9)
BCLC (%)			
A1	78 (63%)	50 (58%)	28 (74%)
A2	18 (15%)	12 (14%)	6 (16%)
A3	9 (7%)	7 (8%)	2 (5%)
A4	15 (12%)	13 (15%)	2 (5%)
B	4 (3%)	4 (5%)	0 (0%)
Esophageal varices (%)	22 (18%)	15 (17%)	7 (18%)
BMI (kg/m^2^)	25.6 (23.7–28.8)	25.7 (24–29)	24.9 (23.2–27.8)
Diabetes (%)	44 (36%)	31 (36%)	13 (34%)
Previous HCC (already treated) (%)	20 (16%)	12 (14%)	8 (21%)
Preoperative blood tests			
Platelet count (× 100/mm^3^)	148 (99–188)	136 (94–182)	160 (112–213)
Total bilirubin (mg/dL)	0.9 (0.6–1.1)	0.9 (0.6–1.2)	0.7 (0.6–1.0)
AST (U/L)	33 (26–59)	35 (26–70)	30 (25–41)
ALT (U/L)	34 (23–58)	37 (26–70)	26 (20–39)
CHE (U/L)	5973 (5028–702)	5794 (4735–7412)	6636 (5280–8014)
ALP (U/L)	83 (67–108)	88 (69–112)	78 (63–106)
α-foetoprotein (ng/mL)	8 (3–28)	8 (3–27)	6 (3–73)

IQR, interquartile range; HCV, hepatitis C virus; HBV, hepatitis B virus; MASH, metabolic dysfunction-associated steatohepatitis**;** MELD, model for end-stage liver disease; BCLC, Barcelona Clinic Liver Cancer; BMI, body mass index; HCC, hepatocellular carcinoma; AST, aspartate aminotransferase; ALT, alanine aminotransferase; CHE, cholinesterase; ALP, alkaline phosphatase

The CT scans were performed across 47 centers, with 52 (42%) carried out at the institution hosting the study. This resulted in considerable heterogeneity in the acquisition and reconstruction parameters, as detailed in Table 2.

**Table 2:** CT, IOUS and histopathological features

Variable	CT	IOUS	Histopathology
HCC diameter (mm) (median; IQR)	25 (17–36)	25 (19–40)	27 (17–40)
Size ≤ 2 cm	46 (37%)	46 (37%)	44 (36%)
Hypervascular	100 (81%)	–	–
Hypoechogenic appearance	–	70 (56%)	–
Homogeneous aspect	68 (55%)	14 (11%)	–
Nodular appearance	108 (87%)	90 (73%)	–
Capsular interruption	12 (10%)	68 (55%)	97 (78%)
Mosaic appearance	47 (38%)	26 (21%)	–
Nodule-in-nodule appearance	9 (7%)	34 (27%)	–
Satellites	6 (5%)	20 (16%)	27 (22%)
MVI	11 (9%)	45 (36%)	51 (41%)
MI-HCC	28 (23%)	51 (41%)	58 (47%)
MI-HCC (size ≤ 2 cm)	4 (3%)	10 (8%)	17 (14%)
HCC differentiation			
Well differentiated			24 (19%)
Moderately differentiated			73 (59%)
Poorly differentiated			27 (22%)

CT, computed tomography; IOUS, intraoperative ultrasound; IQR, interquartile range; HCC, hepatocellular carcinoma; MVI, high-risk signs of vascular/biliary invasion [for CT and IOUS]/microvascular invasion [for histopathology]; MI-HCC, microinvasive hepatocellular carcinoma

For IOUS evaluation, a T-shaped probe was used in 47 patients (38%), whereas a laparoscopic probe was used in 77 patients (62%).

Table 2 summarizes the CT, IOUS and histology findings. With respect to the characteristics of tumor aggressiveness, satellites and MVI were observed in 6 (5%) and 11 (9%) patients, respectively, based on CT examinations. Consequently, the total number of MI-HCC cases identified via CT imaging was 28 (23%), with 4 (14%) classified as early HCC being within 2 cm in diameter.

On IOUS, satellites were identified in 20 patients (16%), whereas MVI was present in 45 patients (36%). Therefore, a total of 51 cases (41%) of MI-HCC were identified, of which 10 (20%) were classified as early HCC.

Histology confirmed satellites in 27 patients (22%) and MVI in 51 (41%), identifying MI-HCC in 58 patients (47%), of which 17 (14%) were no larger than 2 cm in diameter. Furthermore, histological examination revealed that, 24 patients (19%) had well-differentiated HCC, while 100 (81%) had moderately or poorly differentiated HCC. R0 resection was achieved in 120 patients (98%), with a median margin of 5 mm (IQR: 2–25 mm).

Logistic regression analysis demonstrated that several CT and IOUS features significantly correlated with the histopathological presence of MI-HCC (Table 3). Among these factors, the presence of satellites and high-risk signs of vascular/biliary infiltration exhibited the strongest associations with MI-HCC, with ORs as high as 8.71 (*p* < 0.001). Capsular interruption on CT also emerged as a significant predictor (OR = 4.38, *p* = 0.019), whereas a homogeneous lesion was associated with a significantly lower likelihood of MI-HCC (OR = 0.46, *p* = 0.035). Notably, all CT findings correlated with MI-HCC had low sensitivity (< 20%) but very high specificity (> 90%).

**Table 3:** Imaging findings associated with histopathological MI-HCC according to logistic regression analysis

Findings	SE	SP	PLR	NLR	ACC	AUC (95%CI)	OR (95%CI)	p-value
*CT*								
Diameter < 2 cm	31%	58%	0.73	1.20	45%	0.50 (0.40–0.60)	0.62 (0.29–1.27)	0.189
Hypervascular	78%	17%	0.93	1.34	45%	0.54(0.42–0.67)	0.69 (0.28–1.69)	0.420
Homogeneous aspect	45%	36%	0.70	1.52	40%	0.67 (0.52–0.82)	0.46 (0.22–0.95)	**0.035**
Nodular appearance	86%	12%	0.98	1.14	47%	0.55 (0.45–0.66)	0.86 (0.30–2.50)	0.782
Capsular interruption	17%	95%	3.79	0.87	59%	0.69 (0.50–0.88)	4.38 (1.26–20.30)	**0.019**
Mosaic appearance	43%	67%	1.29	0.85	56%	0.70 (0.56–0.84)	1.52 (0.73–3.16)	0.263
Nodule-in-nodule appearance	10%	95%	2.28	0.94	56%	0.66 (0.55–0.77)	2.42 (0.61–11.92)	0.212
Satellites	9%	98%	5.69	0.93	56%	0.50 (0.40–0.60)	6.13 (0.95–119.30)	**0.057**
MVI	17%	97%	5.69	0.85	60%	0.67 (0.52–0.82)	6.67 (1.66–44.69)	**0.006**
*IOUS*								
Diameter ≤ 2 cm	38%	64%	1.04	0.98	52%	0.50 (0.47–0.68)	0.53 (0.25–1.11)	**0.091**
Hypoechogenic appearance	53%	41%	0.90	1.14	47%	0.52 (0.42–0.62)	1.18 (0.58–2.42)	0.648
Homogeneous aspect	12%	83%	0.72	1.06	50%	0.55 (0.40–0.70)	0.66 (0.21–1.91)	0.447
Nodular appearance	71%	26%	0.95	1.14	47%	0.60 (0.49–0.71)	0.43 (0.19–0.96)	**0.039**
Capsular interruption	64%	53%	1.36	0.68	58%	0.53 (0.43–0.63)	0.79 (0.39–1.61)	0.514
Mosaic appearance	19%	77%	0.83	1.05	50%	0.53 (0.40–0.65)	0.81 (0.33–1.94)	0.642
Nodule-in-nodule appearance	29%	73%	1.07	0.97	52%	0.73 (0.62–0.84)	2.01 (0.91–4.55)	**0.086**
Satellites	14%	82%	0.76	1.05	50%	0.59 (0.47–0.70)	8.71 (2.72–38.98)	** < 0.001**
MVI	43%	70%	1.42	0.82	57%	0.69 (0.59–0.79)	5.02 (2.31–11.45)	** < 0.001**

*P*-values < 0.10 are indicated in bold

CT, computed tomography; IOUS, intraoperative ultrasound; SE, sensitivity; SP, specificity; PLR, positive likelihood ratio; NLR, negative likelihood ratio; ACC, accuracy; AUC, area under the receiver operator characteristics curve; OR, odd ratio; MVI, high-risk signs of vascular/biliary microinvasion; MI-HCC, microinvasive hepatocellular carcinoma

In the IOUS subgroup, a nodular appearance was linked to a decreased risk of MI-HCC (OR = 0.43, *p* = 0.039), whereas a lesion size ≤ 2 cm (OR = 0.53, *p* = 0.091) and a nodule-in-nodule appearance (OR = 2.01, *p* = 0.086) presented trends toward a reduced or increased risk, respectively, although with borderline significance. Compared with their CT counterparts, IOUS features demonstrated higher sensitivity but lower specificity.

Based on the predetermined split, 86 patients were used for model development, with 69 allocated for training and 17 allocated for validation. The remaining 38 patients were reserved for independent model testing. Following dimensionality reduction and feature selection, a single SVM model with significant performance was developed using RFs extracted directly from the arterial phase images. The model incorporated four 3D shape RFs (surface volume ratio, flatness, sphericity, maximum 2D diameter row), one first-order RF (skewness), one GLCM RF (joint energy), three GLSZM RFs (small area low gray level emphasis, size zone nonuniformity, zone variance) and one GLDM RF (dependence entropy). No significant models were derived from other contrast phases or from RFs normalized for the liver parenchyma.

**Fig. 3:**
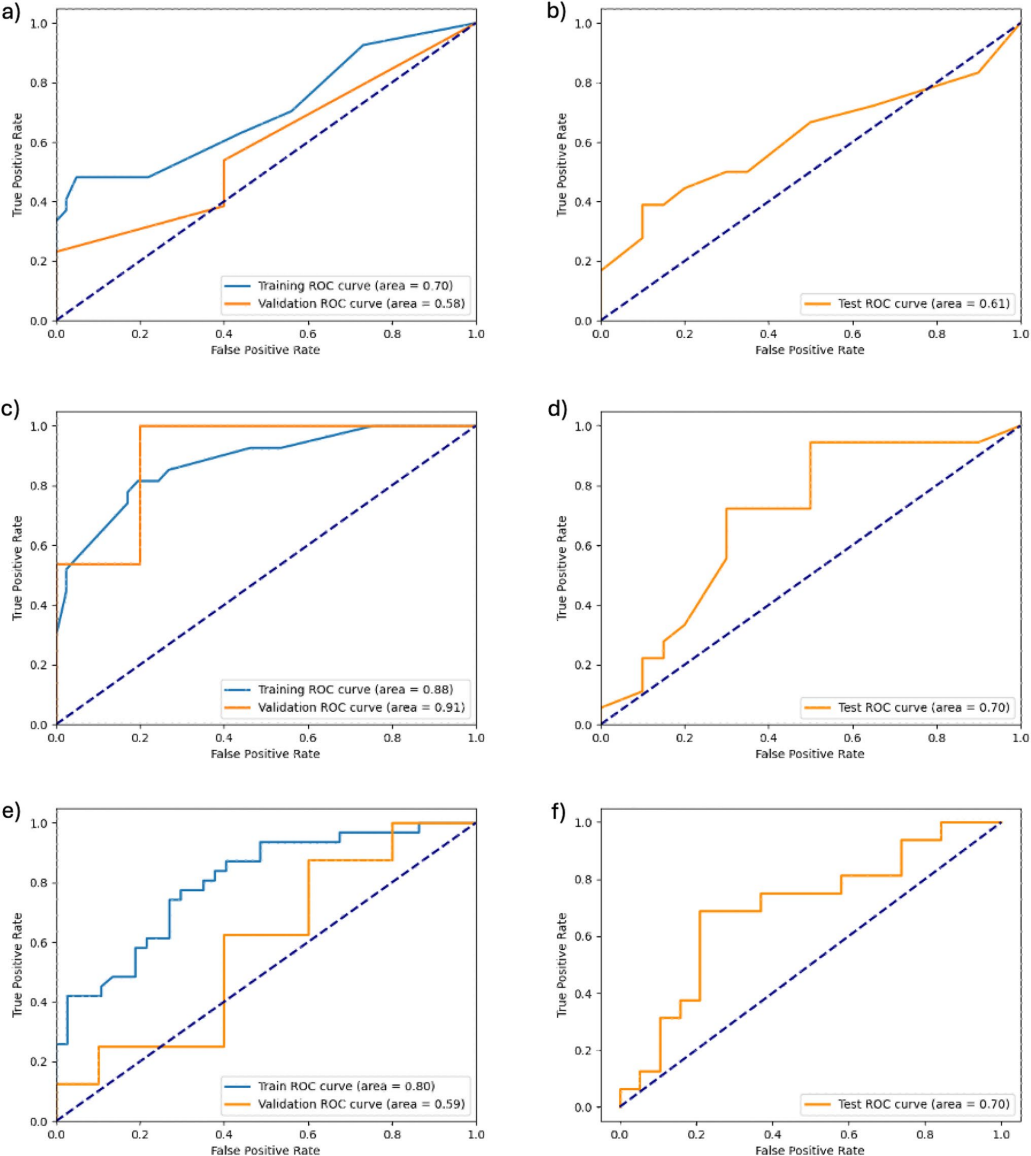
Receiver operating characteristic curve of the SVM models obtained from CT (**a**–**b**), IOUS (**c–d**) and radiomics (**e**–**f**). The left panels (**a**–**c**–**e**) illustrate the performance of the SVM model in the training and validation cohorts, while the right panels (**b**–**d**–**f**) display the model performance in the independent test cohort

When tested on the independent set, the radiomic model yielded an AUC of 0.70 (95%CI: 0.52–0.88; *p* = 0.030). The SVM model built with the previously identified IOUS features, when applied to the same independent test cohort, achieved an AUC of 0.70 (95%CI: 0.51–0.87; *p* = 0.020), whereas the SVM model built with the conventional radiological CT features yielded an AUC of 0.61 (95%CI: 0.42–0.79), which did not reach statistical significance (*p* = 0.117). Details about the performance of the SVM models for the training, validation and test sets are presented in Table 4 and Fig. 3. According to the DeLong test, the performances of the three models did not significantly differ (*p* values ranging from 0.896 to 0.948).

**Table 4:** Performance of the support vector machine (SVM) models for CT, IOUS and radiomics

		SE	SP	PPV	NPV	PLR	NLR	ACC	AUC (IC95%)	*p*-value
CT	Training	48%	95%	87%	74%	9.9	0.5	76%	0.70 (0.56–0.82)	0.001
	Validation	15%	100%	100%	31%	–	0.8	39%	0.59 (0.29–0.83)	0.294
	Test	44%	80%	67%	62%	2.2	0.7	63%	0.61 (0.42–0.79)	0.117
IOUS	Training	70%	85%	76%	81%	4.8	0.3	79%	0.88 (0.78–0.95)	< 0.001
	Validation	54%	80%	88%	40%	2.7	0.6	61%	0.90 (0.63–1.00)	< 0.001
	Test	67%	70%	67%	70%	2.2	0.5	68%	0.70 (0.51–0.87)	0.020
Radiomics	Training	61%	73%	66%	69%	2.3	0.5	68%	0.80 (0.82–1.00)	< 0.001
	Validation	25%	80%	50%	57%	1.3	0.9	56%	0.59 (0.32–0.86)	0.530
	Test	63%	74%	67%	70%	2.4	0.5	69%	0.70 (0.52–0.88)	0.030

SE, sensitivity; SP, specificity; PPV, positive predictive value; NPV, negative predictive value; PLR, positive likelihood ratio; NLR, negative likelihood ratio; ACC, accuracy; AUC, area under the receiver operator characteristic curve

## Discussion

To our knowledge, this is the first study to directly compare IOUS, conventional CT interpretation and CT-based radiomics for the preoperative detection of MI-HCC. This comprehensive, head-to-head comparison provides a unique perspective on the relative strengths of each modality in assessing tumor aggressiveness. Our findings show that both IOUS and radiomics achieved comparable diagnostic performance, highlighting their potential roles as decision support tools in the planning of HCC treatment.

Preoperative identification of MVI, a hallmark of MI-HCC, is critical for tailoring treatment and optimizing outcomes because MVI significantly impacts disease-free survival following curative HR [4, 28–31]. However, as a strictly histological finding [19, 32], MVI poses diagnostic challenges preoperatively, with liver biopsy remaining controversial because of its risks and potential inaccuracies [33, 34]. Recent efforts have focused on improving imaging techniques for MVI detection [34–36]. For instance, Reginelli et al. [15] reported that MVI-positive patients had a larger mean tumor size, but small size does not preclude aggressiveness. In our series, histology revealed MI-HCC in 47% of patients, with nearly one-third of tumors measuring < 2 cm, underscoring the need for reliable early-stage predictors of MI-HCC beyond tumor size.

Consistent with the literature [15], we found that capsule interruption on preoperative CT increased MI-HCC risk fourfold, indicating high specificity but limited sensitivity. Additionally, homogeneous HCC appearance was associated with reduced MI-HCC risk, likely reflecting uniform tumor growth with minimal tissue disruption [37, 38]. However, well-defined margins, nodule-in-nodule appearance and mosaic patterns did not correlate with MI-HCC in our study, likely due to variability in interpretation or CT acquisition protocols. These findings highlight the limitations of conventional CT for reliable MI-HCC prediction.

Unlike other modalities, IOUS provides real-time imaging to assess aggressive HCC behavior during surgery [8]. A prospective study by Santambrogio et al. [10] demonstrated strong concordance between histology and IOUS-detected features, including satellite nodules and signs of vascular or biliary invasion, even in early-stage HCC. These results highlight IOUS as a critical tool for identifying high-risk HCC phenotypes, bridging imaging and histopathology. In our study, these features were confirmed as independent risk factors for MI-HCC. Additionally, irregular margins and nodule-in-nodule appearance are associated with this phenotype, indicating that IOUS has superior sensitivity for detecting intralesional patterns compared with CT.

Because performance metrics for both CT and IOUS features suggest that no single variable can reliably identify MI-HCC, radiomics and machine learning have emerged as promising approaches to extract meaningful information from medical images [39]. Our radiomic model yielded an AUC of 0.70, which is lower than the reported range of 0.58–0.90 in other studies [5, 12, 13, 40, 41]. Such discrepancies may arise from differences in feature extraction, region of interest selection, tumor size, the number and type of radiomic features, and the techniques employed to minimize overfitting [40]. Indeed, radiomics is known to be susceptible to inflated results, especially when no feature selection is performed or when no independent samples are tested. To reduce overfitting, we used a rigorous selection process involving the exclusion of autocorrelated features and the application of LASSO regularization. Moreover, our dataset included CT scans with heterogeneous acquisition and reconstruction settings, probably reducing the model performance but ensuring that only features robust to these variations were ultimately incorporated. By evaluating each contrast phase separately before model building, we also avoided overloading the model with excessive features, ultimately selecting a single model based solely on the arterial phase.

Compared with Renzulli et al. [13], we used an IBSI-compliant tool for RFs extraction, ensuring greater transparency and reproducibility. Notably, both studies highlighted “skewness” as a significant RF among the identified variables. A skewed intensity histogram suggests nonuniform perfusion within the lesion, likely attributable to tumor MVI. Similarly, the “joint energy,” which quantifies the uniformity of intensity textures, likely captures the effects of disrupted arterial supply and the heterogeneous architecture of the local tumor environment. The selected shape RFs (surface-to-volume ratio, flatness and sphericity) may indicate irregular tumor margins and distortion, reflecting how the tumor infiltrates along microvascular structures or fibrous septa. The “small area low gray level emphasis” quantifies small, low-intensity homogeneous regions, potentially corresponding with areas of necrosis, fibrosis or reduced blood flow. Similarly, the “size zone nonuniformity” and “zone variance” describe variability in the size of homogeneous zones, likely reflecting a patchy infiltration pattern, with areas that may be densely packed with neoplastic cells and abnormal vessels, whereas others are sparser or fibrotic. Ultimately, the “dependence entropy” reflects intensity complexity and randomness, likely correlating with a more disorganized tissue structure and chaotic blood supply patterns due to MVI.

Among the SVM-based models, no significant performance differences were observed overall. However, only the IOUS and radiomic models reached statistical significance, highlighting the limitations of conventional CT features in reliably detecting MI-HCC. These shortcomings are likely derived from the lower spatial and contrast resolution of conventional CT.

Although the radiomics model demonstrated effectiveness in predicting microvascular invasion in HCC, the independent test cohort was relatively small, and the AUC values of both the radiomics and IOUS models were lower than those reported in several previous radiomics studies on MI-HCC. Importantly, the use of an independent test cohort reflects a deliberate methodological choice prioritizing robustness and generalizability over performance optimization. Accordingly, the aim of this study was to evaluate radiomics as an adjunctive tool rather than as a stand-alone diagnostic approach. Preoperative assessment of MI-HCC remains an unresolved clinical challenge, and radiomics may provide added value only when integrated within a multidisciplinary team decision-making framework.

Our study has several limitations, the first being its retrospective design, which introduces a risk of selection bias. Additionally, we included a 3-mm rim of perilesional liver parenchyma in the radiomic analysis because evidence suggests that incorporating the peritumoral area can enhance the prediction of MI-HCC [5, 12, 13, 42]. However, the optimal definition of this peritumoral area remains unclear, with studies suggesting a range from 3 mm to 1 cm beyond the tumor margins. To reduce the risk of including confounding structures, we selected a smaller peritumoral region, minimizing the need for post hoc corrections but potentially limiting predictive power.

Notably, radiomic features normalized to background liver parenchyma did not yield significant models, suggesting limited robustness of ratio-based normalization in this context. In cirrhotic patients, parenchymal heterogeneity makes a single reference ROI an unstable baseline: Measurements can vary with ROI size and placement, and this variability propagates into the normalized features, effectively amplifying noise and attenuating tumor-specific signal. More robust approaches, such as averaging multiple parenchymal ROIs, using whole-liver parenchyma segmentation, and/or harmonizing features across scanners, may be required to improve performance.

Another important limitation of this study is the relatively small sample size, particularly given the division into training, validation and independent test cohorts for model development and evaluation. This subdivision inevitably reduces statistical power and may constrain the generalizability of the findings. However, it is important to note that the primary objective of this study was not to establish absolute performance metrics for radiomics, but rather to evaluate its diagnostic value within a comparative framework alongside IOUS and conventional CT interpretation. To mitigate the risk of overfitting and enhance model robustness, we implemented rigorous dimensionality reduction techniques. Additionally, the inclusion of CT scans from nearly 50 different institutions introduced substantial acquisition heterogeneity, which challenged the stability of radiomic features and ensured that only reproducible, generalizable features contributed to the final model. Together, these methodological safeguards support the credibility of the comparative conclusions, even within the context of a limited sample size. Moreover, we employed SVM due to its robustness with small datasets; however, alternative algorithms such as deep learning or ensemble methods may have further improved model performance. Nonetheless, the interpretability and generalizability of more complex algorithms would require careful validation, especially in the context of limited data. Prospective multicenter studies with standardized imaging protocols and broader exploration of machine learning strategies will be essential to validate and expand the applicability of our findings.

## Conclusion

Although conventional CT imaging did not achieve statistically significant diagnostic performance, preoperative CT-derived radiomics demonstrated accuracy comparable to that of IOUS in the assessment of MI-HCC. Despite the stringent workflow, which may have limited overall model performance, the identified radiomic features suggest a specific biological relevance, supporting the potential role of radiomics as a noninvasive tool for preoperative MI-HCC risk stratification when integrated with conventional imaging findings within a multidisciplinary team discussion. With continued advancements and standardization of radiomic workflows, their integration into clinical practice could improve MI-HCC prediction, enabling more personalized decision-making and treatment planning.
